# Similarities in the Biophysical Properties of Spiral-Ganglion and Vestibular-Ganglion Neurons in Neonatal Rats

**DOI:** 10.3389/fnins.2021.710275

**Published:** 2021-10-12

**Authors:** Radha Kalluri

**Affiliations:** Caruso Department of Otolaryngology-Head and Neck Surgery, Zilkha Neurogenetics Institute, Keck School of Medicine of University of Southern California, Los Angeles, CA, United States

**Keywords:** ion channels, voltage threshold, vestibular ganglion neurons, spiral ganglion neurons, firing patterns, current threshold, neuronal excitability, biophysical properties

## Abstract

The membranes of auditory and vestibular afferent neurons each contain diverse groups of ion channels that lead to heterogeneity in their intrinsic biophysical properties. Pioneering work in both auditory- and vestibular-ganglion physiology have individually examined this remarkable diversity, but there are few direct comparisons between the two ganglia. Here the firing patterns recorded by whole-cell patch-clamping in neonatal vestibular- and spiral ganglion neurons are compared. Indicative of an overall heterogeneity in ion channel composition, both ganglia exhibit qualitatively similar firing patterns ranging from sustained-spiking to transient-spiking in response to current injection. The range of resting potentials, voltage thresholds, current thresholds, input-resistances, and first-spike latencies are similarly broad in both ganglion groups. The covariance between several biophysical properties (e.g., resting potential to voltage threshold and their dependence on postnatal age) was similar between the two ganglia. Cell sizes were on average larger and more variable in VGN than in SGN. One sub-group of VGN stood out as having extra-large somata with transient-firing patterns, very low-input resistance, fast first-spike latencies, and required large current amplitudes to induce spiking. Despite these differences, the input resistance per unit area of the large-bodied transient neurons was like that of smaller-bodied transient-firing neurons in both VGN and SGN, thus appearing to be size-scaled versions of other transient-firing neurons. Our analysis reveals that although auditory and vestibular afferents serve very different functions in distinct sensory modalities, their biophysical properties are more closely related by firing pattern and cell size than by sensory modality.

## Introduction

Auditory and vestibular afferents are bipolar neurons that receive, filter, and transmit information from the sensory epithelium to the brainstem. In both systems, the afferents are each comprised of functionally distinct neuronal sub-groups that convey different qualities of sensory information to the brainstem (e.g., rapid and slow head movements in the vestibular system; or sound intensity in the auditory system). Previous work in auditory and vestibular physiology classified neuronal sub-groups by their dendritic morphology, synaptic specializations, and patterns of connectivity with hair cells to account for these parallel information pathways.

**FIGURE 1:**
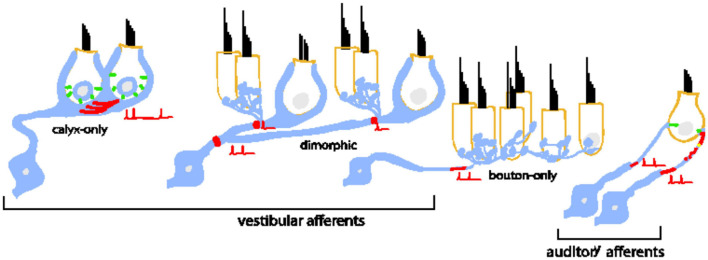
Bipolar neurons of the inner ear are diverse in their morphology, dendritic arborization, and patterns of connectivity with inner hair cells. Type I spiral ganglion afferents in the auditory nerve have a single dendritic terminal that receives input from a single ribbon synapse at an inner hair cell. An individual inner hair cell has many synaptic ribbons that provide input to many Type I spiral ganglion neurons with a wide range of sound intensity thresholds. Vestibular afferents have diverse dendritic morphologies. Some neurons form post-synaptic calyx terminals that envelop the entire basal pole of Type I vestibular hair cells. Other neurons branch extensively to make many bouton terminals on Type II hair cells. A large group of neurons is dimorphic because the neurons form both calyceal and bouton terminals on Type I and Type II hair cells. The prevalence of dendritic morphology changes based on epithelial zone. Pure calyx afferents tend to be found in the central zones of vestibular epithelia where irregular-timing spiking is prevalent *in vivo*. Bouton afferents are found in the peripheral zones where regular-timing spiking is prevalent. Dimorphic afferents are found throughout. In both bipolar neuron groups, spike initiation occurs on the dendrite near the terminals.

When considered across the two systems, inner-ear afferents are remarkably diverse in dendritic and terminal morphologies (Figure 1). Some vestibular afferents make spatially compact connections by forming giant cup-like (calyx) terminals around the entire basal pole of one or more hair cells, while others form large spatially extended dendritic arbors that contact hundreds of hair cells with varying combinations of calyces and tiny bouton-like terminals (e.g., Goldberg et al., 1990). In contrast to the varied combination of inputs driving vestibular afferents, the Type I auditory neurons (the primary cell type in the auditory nerve) make a single bouton-like connection opposing a single synaptic ribbon (Liberman, 1982).

**FIGURE 2:**
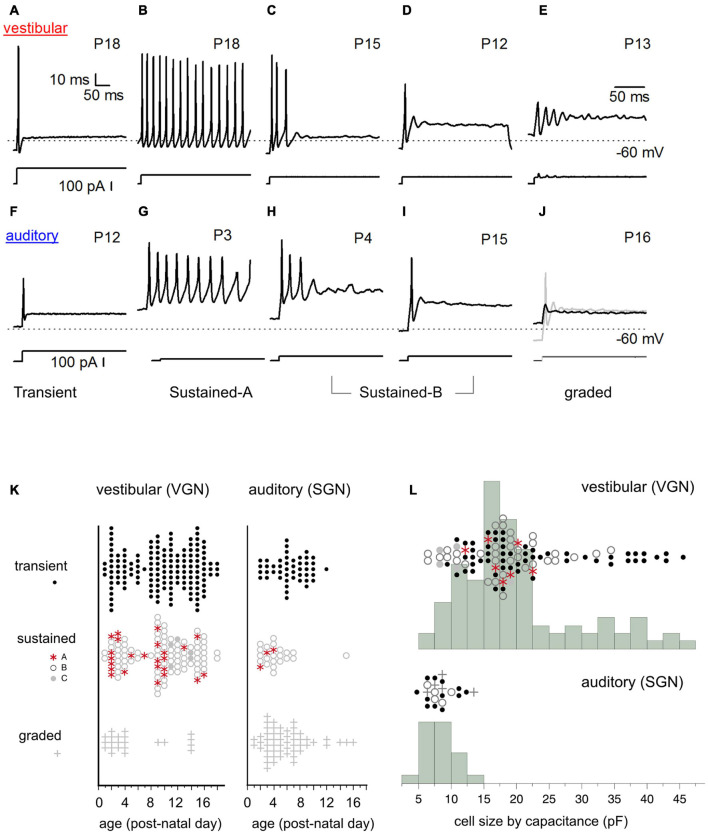
Diversity of *in vitro* firing patterns in response to step current injection. **(A–E)** Firing patterns in five example vestibular ganglion neurons. Transient-spiking **(A)**, sustained-A spiking **(B)**, sustained-B spiking **(C,D)**. Non-spiking/graded responses **(E)**. Dashed line indicates –60 mV. Traces below the firing patterns indicate stimulus. **(F–J)** Similar range of firing patterns in five example spiral ganglion neurons (auditory). Transient-spiking **(F)**, sustained-A spiking **(G)**, sustained-B spiking **(H,I)**. Non-spiking/graded response (black trace). **(J)** Spiking is induced by injecting a –10 pA hyperpolarizing current before applying the current step (gray trace) **(J)**. **(K)** Prevalence of firing patterns as a function of postnatal age in VGN (left) and SGN (right). **(L)** Distribution of somatic cell size in vestibular and spiral ganglion. Cell capacitance values were available in fewer cells than shown in **(K)**. In **(K,L)**, symbols indicate firing patterns as follows: Transient (black dots), sustained-A (red asterisks), sustained-B (open gray circles), non-spiking plus graded (plus sign).

Despite the many morphological and functional differences between auditory and vestibular afferents, many of the individual components of their synapses are similar. For example, in both systems, sensory hair cells bearing synaptic ribbons (Merchan-Perez and Liberman, 1996; Lysakowski and Goldberg, 2008) release glutamate to drive post-synaptic glutamate receptors (Glowatzki and Fuchs, 2002; Songer and Eatock, 2013; Sadeghi et al., 2014). Spiking is likely triggered at ion channel dense hemi-nodes located on peripheral dendrites close to the sensory epithelium (see red shading in Figure 1 schematizing the putative spike initiation zone) (Hossain, 2005; Lysakowski et al., 2012). Most relevant to this paper is that *in vitro* studies have shown that the neurons are biophysically diverse in both systems. Some neurons respond only to the onset of current injections (temporal differentiation), while others also respond to the continuous portion of the stimulus (temporal integration) (described further in Figure 2). Such diversity allows the neuronal subgroups to encode specific features of the incoming sensory information.

Biophysical diversity arises from differences in ion channel composition. Across many studies in both systems, gradients in the same two classes of ionic currents – low-voltage gated potassium currents driven by Kv1 and KCNQ channels (*I*_*KL*_) and hyperpolarization-activated cationic currents conducted through HCN channels (*I*_*h*_) – are proposed as being crucial for driving biophysical diversity (Mo and Davis, 1997a,b; Mo et al., 2002; Iwasaki et al., 2008; Kalluri et al., 2010; Yi et al., 2010; Almanza et al., 2012; Yoshimoto et al., 2015). Despite the many qualitative similarities between the intrinsic properties of vestibular- and spiral ganglion neurons, there are no direct comparisons between the two groups. Here i take the first steps by comparing firing patterns and neuronal excitability from patch-clamp recordings made in our laboratory from spiral- and vestibular-ganglion neurons.

## Materials and Methods

### Preparation

Patch-clamp recordings were made from spiral-ganglion somata in semi-intact cochlear whole-mount preparations and vestibular-ganglion somata from disassociated neurons. Neuron preparations were from Long-Evans rats from either sex ranging in age from postnatal day (P)1 through P18.

Animals were handled according to the National Institutes of Health Guide for the Care and Use of Laboratory Animals. All procedures were approved by the animal care committee of the Massachusetts Eye and Ear Infirmary, The House Research Institute, or the University of Southern California.

The data presented have appeared in previous publications. Readers interested in detailed descriptions of the methods can refer to the original manuscripts (Kalluri et al., 2010; Ventura and Kalluri, 2019; Markowitz and Kalluri, 2020). The following is a summary.

Cochlear and vestibular ganglion were bathed in and perfused throughout the dissection and recording session with Liebovitz medium supplemented with 10 mM HEPES and titrated with NaOH to 7.35 pH (L-15).

*Spiral Ganglion*: Cochlear middle turns were dissected from the middle turn and mounted as whole-mount epithelia under nylon threads on a glass coverslip as previously described in Markowitz and Kalluri (2020). The preparations were treated with an enzymatic cocktail containing L-15, 0.05% collagenase, and 0.25% trypsin for 15–20 min at 37 degrees C and subsequently perfused with fresh oxygenated L-15 during the entire recording. Recordings were made from the cell bodies (somata) of the bipolar spiral ganglion neurons. The semi-intact preparation contained the peripheral dendrites of the bipolar neurons and their hair cell connections. Recordings began and ended between 1 and 5 h after dissection.

*Vestibular Ganglion*: The superior vestibular ganglion was dissected, dissociated, and cultured as previously described (Kalluri et al., 2010; Ventura and Kalluri, 2019). Briefly, vestibular ganglia were treated with the same trypsin/collagenase cocktail described above for spiral ganglion. Enzymatic incubation lasted between 20 and 40 min, depending on age. Ganglia were passed through a series of fire-polished Pasteur pipettes to dissociate cells mechanically. Disassociated cells were plated onto coated coverslips (poly-d-lysine coated glass-bottomed culture dishes or polyethlylamine coated coverslips) and cultured overnight in DMEM with 10 mM HEPES, penicillin-streptomycin, 5% FBS, as previously described.

Enzymatic digestion helps to remove the myelin layers that wrap around the somata of spiral and vestibular ganglion neurons. Demyelination allows electrodes to access the neuronal membrane for patch-clamp recording. Some enzymes are known to impact the function of ion channels (Quandt, 1987; Armstrong and Roberts, 1998). For example, papain distorts the inactivation properties of BK channels by cleaving their extracellular domains (Armstrong and Roberts, 1998). Here we treat both the VGN and SGN with the same enzymatic cocktail containing trypsin and collagenase, thus providing consistency across the two systems. In both systems, the incubation time in enzyme was empirically titrated at each age range to maximize cell yield.

### Electrophysiology

Cochlear preparations were viewed at X630 using a Zeiss Axio-Examiner D1 microscope fitted with Zeiss W Plan-Aprochromat optics. Vestibular ganglion preparations were viewed at X430 on an inverted microscope fitted with DIC optics (e.g., Zeiss Axiovert 135). Signals were driven, recorded, and amplified by either an Axoclamp 200B or Multiclamp 700B amplifier, Digidata 1440 board, and pClamp software.

Recording pipettes were fabricated using filamented borosilicate glass. Pipettes were fired polished to yield an access resistance between 5 and 7 MΩ. The tip of each recording pipette was covered in a layer of parafilm or sylgard to reduce pipette capacitance. Cochlear recordings were made in ruptured patch mode with recording pipettes filled with the following standard internal solution (in mM): 135 KCl, 3.5 MgCl_2_, 3 Na_2_ATP, 5 HEPES, 5 EGTA, 0.1 CaCl_2_, 0.1 Li-GTP, 100 cAMP, and titrated with KOH to a pH of 7.35. This yielded a total potassium concentration of 165 mM with a total osmolality of 300 mmol/kg.

Vestibular ganglion recordings were made in either ruptured-patch mode with a similar standard internal solution (see above) or in perforated-patch mode. The perforated-patch internal solution contained (in mM): 75 K_2_SO_4_, 25 KCl, 5 MgCl_2_, 5 HEPES, 5 EGTA, 0.1 CaCl_2_, and titrated with 1M KOH to a pH of 7.4. Amphotericin B (240/ml, 158 Sigma-Aldrich) was dissolved in DMSO and added to the perforated patch solution on the day of recording. I pooled the data from perforated and ruptured-patch recordings as the gross electrophysiological features were not dependent on the recording mode. Voltages are reported without correcting a junction potential of approximately 3.8 mV in ruptured-patch or 5.5 mV in perforated-patch (calculated by JPCalc as implemented in pClamp 10.7, Barry 1994).

### Analysis

Spike features were analyzed from current-clamp recordings in response to families of depolarizing current steps. Current steps were either 200 or 400 ms in duration and incremented from −120 to 200 pA in 10 pA increments. In some cells, current injection levels were increased to 400 pA. Cells were first qualitatively classified based on the pattern of spike accommodation in response to injected currents. I characterized resting potential, cell size as inferred from capacitance, input resistance near resting potential, current threshold, voltage threshold, and first spike latency.

*Resting potential* (*V*_*rest*_, mV) was estimated as the cell membrane potential averaged before step current injection.

*Input resistance* (*R*_*in*_, GOhm) was calculated as the slope of the voltage-current relationship produced 100 ms after a ± 10-pA step current injection.

*Membrane capacitance* (*C*_*m*_, pF) was estimated in voltage clamp from the built-in membrane test protocol in pClamp and capacitance estimation circuitry of the amplifier. Note that this is different from our previous papers where we estimated *C*_*m*_ from current-clamp recordings by fitting an exponent to the voltage response for a small hyperpolarizing current step. However, measuring capacitance in current clamp can lead to errors in capacitance estimation for spatially extended neurons and if the activation of voltage-gated channels distorts the time course of membrane charging. Because of this potential for error, we only report cell capacitance when it was measured in voltage clamp. Note also that this resulted in fewer cells in which we have *C*_*m*_ estimates than cells in which we recorded firing patterns.

*Voltage Threshold (V*_*thresh*_, *mV).* Voltage threshold was the membrane voltage *V*_*m*_ at which d*V*_*m*_/d*t* changes rapidly. A 5 mV/ms threshold criterion was suitable for detecting action potentials in both vestibular and auditory ganglion neurons. The criterion value was defined to reliably detect spikes while avoiding graded depolarizations and spurious depolarizations due to capacitance artifacts and noise. Some cells, like the graded-firing group in SGN and sustained-C group in VGN, were excluded from subsequent analysis because their stimulus-induced depolarizations did not meet the criterion value.

*Relative Voltage Threshold (δV_*th*_, mV)* is the depolarization needed relative to resting potential to initiate an action potential. δ*V*_*th*_ = *V*_*thresh*_—*V*_*rest*_.

*Current Threshold* (*I*_*thresh*_, pA) was defined as the minimum current required (in 10 pA increments) to produce an action potential.

*Response Latency (L, ms)* was defined as the delay between the onset of stimulus current and the peak of the first action potential induced by the stimulus at current threshold.

*Net Excitability (mV/pA or GOhms)*: The ratio of relative voltage threshold to current threshold (*V*_*thresh*_-*V*_*rest*_)/*I*_*thresh*_

*Charge Threshold (Q, femto Coulombs)*: The charge threshold was defined as the product between the current threshold and first spike latency. For a step current injection, this approximates the charge delivered to evoke the first action potential.

#### Statistical Analysis

pClamp software was used to gather raw data from electrophysiological recordings. MATLAB was used to batch process and uniformly quantify the firing pattern features from all recordings. Statistical analysis was performed in JMP software package. Student’s *t*-test was used to compare the means of two distributions with equal variance (as determined by Levine’s test). Regression slope analyses are reported in the figure captions. One-way analysis of covariance was used to compare groups when one covariate was a continuous variable (e.g., when comparing resting potential vs. age in firing-pattern groups). *Post-hoc* Tukey HSD tests compared the least squared adjusted means. The results of the ANCOVA analyses are shown in Table 1. All the individual data points with regressions and 95% confidence intervals are shown in figures. An alpha level of 0.05 was used for all statistical tests.

**TABLE 1:** Analysis of covariance.

	Source	Nparm	DF	Sum of squares	F ratio	Prob > F
Figure 3	Ganglion	1	1	732.2971	12.1165	0.0006
*V*_*rest*_ (mV)	Age (post-natal days)	1	1	3463.6387	57.3088	<0.0001
	Ganglion*age (post-natal days)	1	1	10.2708	0.1699	0.6806
Figure 3A *V*_*rest*_	Age (post-natal days)	1	1	1981.3045	35.2857	<0.0001
Transient	Ganglion (VGN/SGN)	1	1	545.8877	9.7219	0.0023
	Age*ganglion	1	1	13.395	0.2386	0.6261
Figure 3B *V*_*rest*_	Age (post-natal days)	1	1	1459.5773	22.6073	<0.0001
Sustained (a+b)	Ganglion	1	1	3.3321	0.0516	0.8209
	Ganglion*age (post-natal days)	1	1	7.7828	0.1205	0.7293
Figure 3C *V*_*rest*_	Age of animal (post-natal days)	1	1	1603.5887	48.311	< 0.0001
Graded	Ganglion	1	1	536.4468	16.1614	0.0001
	Ganglion*age (post-natal days)	1	1	1.0542	0.0318	0.8591
Figure 4A	Ganglion	1	1	299.4174	6.03	0.0151
*V*_*thresh*_ (mV); *n* = 164	*V*_*rest*_, (mV)	1	1	2965.569	59.7241	<0.0001
	Ganglion*V_*rest*_, (mV)	1	1	0.0209	0.0004	0.9837
Figure 4D	*V*_*rest*_, (mV)	1	1	52.32906	0.7889	0.3768
Δ*V*_*thresh*_ (mV)	Ganglion	1	1	315.8627	4.7619	0.0317
Transient; *n* = 93	Ganglion* *V*_*rest*_, (mV)	1	1	0.06481	0.001	0.9751
Figure 4E	*V*_*rest*_, (mV)	1	1	335.095	19.8699	<0.0001
Δ*V*_*thresh*_ (mV)	Ganglion	1	1	29.41228	1.744	0.1911
Sustained (a+b); *n* = 71	Ganglion* *V*_*rest*_, (mV)	1	1	0.004	0.0002	0.9878
Figure 5A	*V*_*rest*_, (mV)	1	1	67150.39	14.0502	0.0003
*I*_*thresh*_ (pA)	Firing Pattern	1	1	233488.9	48.854	<0.0001
VGN	*V*_*rest*_, (mV) * Firing pattern	1	1	25456.27	5.3263	0.0225
Figure 5B	*V*_*rest*_, (mV)	1	1	2750.983	4.7693	0.0325
*I*_*thresh*_ (pA)	Firing pattern	1	1	9951.6	17.2527	<0.0001
SGN	*V*_*rest*_, (mV) * Firing pattern	1	1	494.1974	0.8568	0.358
Figure 5D	*V*_*rest*_, (mV)	1	1	8.577368	47.4505	<0.0001
*Net excitability (gOhm)*	Firing Pattern	1	1	1.05186	5.8189	0.0174
VGN	*V*_*rest*_, (mV) * Firing Pattern	1	1	0.211134	1.168	0.282
Figure 5E	*V*_*rest*_, (mV)	1	1	0.081531	1.7086	0.1988
*Net excitability (gOhm)*	Firing pattern	1	1	0.001529	0.032	0.8589
SGN	*V*_*rest*_, (mV) * Firing pattern	1	1	0.027033	0.5665	0.4562
Figure 6A	*R*_*in*_ *(gOhm)*	1	1	5.439455	42.2084	<0.0001
*Net excitability* (*gOhm*)	Firing pattern	1	1	5.426628	42.1089	<0.0001
	*R*_*in*_ * Firing pattern	1	1	0.071217	0.5526	0.4583
Figure 6B	*C*_*m*_ (pF)	1	1	231.62668	6.6882	0.0110
Response latency	Firing type	1	1	296.73012	8.5681	0.0041
	*C*_*m*_ (pF)*Firing type	1	1	0.26040	0.0075	0.9311

* indicates the interaction between two source variables.

## Results

### Heterogeneity of Firing Patterns

The somata of spiral and vestibular ganglia respond with a wide range of firing patterns to injected currents (Figures 2A–E spiral ganglion, Figures 2F–J vestibular ganglion). We broadly grouped cells based on these firing patterns (readers can find more details about this classification scheme in Kalluri et al., 2010; Markowitz and Kalluri, 2020). Here the same naming convention previously applied to the vestibular ganglion (blue text under the firing patterns) is applied to the spiral ganglion.

I classified neurons into two broad firing pattern groups, transient-firing and sustained-firing. Transient-firing neurons produce a single action potential (spike) at the onset of the current step (Figures 2A,F; VGN and SGN, respectively). For current injections greater than threshold, the firing pattern of most transient-spiking neurons is invariant to intensity. In contrast, sustained-firing neurons vary in the degree of accommodation. Some neurons have slowly accommodating firing patterns with long trains of spikes throughout the depolarizing step (sustained*-*A firing patterns, Figures 2B,G). Other neurons have intermediate degrees of accommodation (sustained-B; Figures 2C,D,H,I, respectively). The degree of accommodation observed in sustained-firing patterns varies from cell to cell and depends on current amplitude.

In both vestibular and spiral ganglion, some cells do not fire robust action potentials. We classified these non-spiking responses as graded (Figures 2E,J). The presence of an action potential was defined as reaching a criterion depolarization rate of 5 mV/ms. We previously classified non-spiking cells with prominent voltage oscillations follow a short first spike or voltage depolarization as sustained-C/resonant (Figure 2E). Here, non-spiking cells with and without voltage oscillations were classified as graded. Notably, non-spiking cells in both the vestibular and spiral ganglion could produce an action potential if they were first hyperpolarized to below their natural resting potential before applying current steps (gray curve in Figure 2J shows an example in spiral ganglion).

Spike patterns were analyzed in 131 spiral ganglion neurons and 294 vestibular ganglion neurons. In both spiral and vestibular ganglion, the different spike patterns were not uniformly prevalent. Most cells producing action potentials had transient-firing patterns (151/294 VGN; Figure 2K, left; 47/131 SGN; Figure 2K, black dots). Within the sustained group (106/294 VGN, 24/131 SGN), we encountered sustained-B firing patterns (76/106 VGN; 21/24 SGN, Figure 2K) more often than sustained-A firing patterns (25/106 VGN, 3/24 SGN). Graded or non-spiking cells formed a significant proportion of the data set in the spiral ganglion (54/131) but were less frequently encountered in vestibular ganglion (22/294 VGN). This could be due to methodological differences because SGN recordings were from semi-intact acute preparations. In contrast, the VGN recordings were from dissociated cultured preparations (see section “Discussion” on this point).

The prevalence of different firing-pattern groups changes with development, with the number of sustained-A firing neurons decreasing with postnatal age in both VGN and SGN. To illustrate the age-dependent change in firing pattern prevalence, we grouped the data as < P10 and ≥ P10. Below P10, 16 out of 48 sustained-spiking responses in VGN (∼33%) were sustained-A patterns. After P10, 8 out of 54 sustained-spiking responses (∼15%) were sustained-A firing patterns. In spiral ganglion, the dataset is much more limited in terms of postnatal age, so we did not quantify prevalence in a similar way. However, only 3 out of 23 sustained-spiking responses were classified as sustained-A; all three observations were recorded before P4.

#### Cell Size

One of the most striking differences between vestibular and spiral ganglion neurons is somatic size (as inferred from the membrane capacitance estimates from voltage-clamp measurements). Vestibular neurons have a wide range of membrane capacitance, ranging from 5 to 45 pF (Figure 2L). Note that the number of cells in which capacitance measurements were available for comparison is fewer than we measured firing patterns (104/294 in VGN and 23/131 in SGN). This is because the present analysis was limited to those cells in which capacitance measurements were made in voltage-clamp. This constraint was applied because voltage-clamp based capacitance estimates are less likely to be distorted voltage gated currents like *I*_*KL*_ or by the extended neuronal morphology in the semi-intact spiral ganglion preparation (Golowasch et al., 2009).

The distribution of cell size is bimodal in VGN (Figure 2L, top). Most VGN (∼80%) are small to intermediate-sized cells with capacitance values ranging between 5 and 25 pF. Both transient-firing and sustained-firing cells are found in the first mode (Figure 2L; firing type is indicated by the same symbols as defined in Figure 2K). The remaining ∼20% of VGN are large cells with capacitance values between 30 and 45 pF. In this size range, transient-firing patterns are the dominant group (16/21 cells, Figure 2L).

In contrast to the wide range of cells sizes seen in vestibular ganglion, spiral ganglion somata are smaller and more homogeneous in cell size (with capacitance values ranging between 6 and 10 pF; Figure 2L, bottom). Based on confocal scans in the neonatal rat spiral ganglion, Markowitz and Kalluri (2020) estimated that the SGN reported in this study had average diameters around 12.7 ± 0.3 μm, *n* = 28. These values are consistent with measurements made in other mammalian species where the mature somatic diameters of SGN range between 10 and 15 μm (Romand and Romand, 1990; Berglund and Ryugo, 1991; Tsuji and Liberman, 1997; Nayagam et al., 2011). Assuming an unmyelinated spherical soma (with a specific capacitance around ∼1 μF/cm^2^), the somatic capacitance would be around 5.1 ± 0.2 pF. Based on the similarities in values between the capacitance as estimated from voltage clamp and estimated cell diameters, we conclude that small capacitance (in comparison to vestibular ganglion) is representative of spiral ganglion neurons. The differences in cell-capacitance between spiral and vestibular ganglion is unlikely to be driven by the difference in the average age of the present comparison.

Overall, somatic size is more variable in vestibular ganglion neurons than it is in spiral ganglion neurons. However, cell size was not a robust predictor of firing pattern in either VGN or SGN, excepting the largest VGN, which are predictably transient-firing.

### Comparing the Excitability of Vestibular and Spiral Ganglion Neurons

I measured resting potential, voltage threshold relative to resting potential, current threshold, and first-spike latency to quantify cell excitability. I examined the covariances between these features, which then compared across vestibular and spiral ganglion to identify similar and different features between the systems.

**FIGURE 3:**
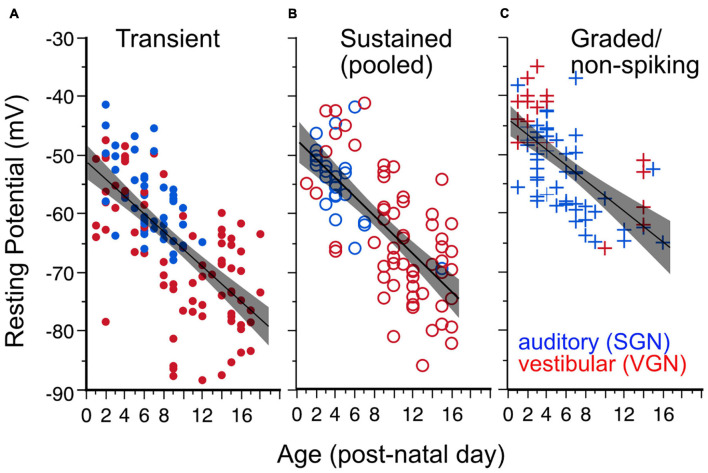
Resting potential hyperpolarizes with age for all firing pattern groups. Lines plus shaded areas show linear regressions plus 95% confidence intervals. Blue symbols indicate spiral ganglion neurons, whereas red symbols indicate vestibular ganglion neurons. Each data point represents the resting potential of individual ganglion neurons. **(A)** Resting potential vs. postnatal age in transient-spiking somata. Y (Resting Potential) = –1 – 1.49*X (post-natal age). *F*(1, 129) = 100.92, *p* = 0.0001. **(B)** Resting potential vs. postnatal age in sustained-spiking neurons. Here sustained-A and sustained-B groups are pooled together and represented by open circles. Y = –47.56 – 1.615*X. *F*(1, 82) = 80.10, *p* ≤ 0.0001. **(C)** Resting potential vs. postnatal age in non-spiking/graded neurons. Y = –44–1.306*X, *F*(1,69) = 45, *p* < = 0.0001.

Resting potential hyperpolarizes with postnatal age (Figure 3) in both spiral and vestibular ganglion (*p* < 0.0001). As illustrated in Figure 3, the age-dependent hyperpolarization in resting potential is evident in all three firing pattern groups (transient-firing Figure 3A, sustained-A + sustained-B pooled Figure 3B, and graded/non-spiking Figure 3C). This hyperpolarization of resting potential is consistent with previous findings in both VGN and SGN showing that maturing ganglion neurons acquire larger potassium currents (Mo et al., 2002; Kalluri et al., 2010; Iwasaki et al., 2012; Markowitz and Kalluri, 2020). The spiral and vestibular ganglion neurons data are plotted together but indicated by color for easy identification (red for VGN and blue for SGN).

The dependence of resting potential on age is similar for both spiral ganglion and vestibular ganglion neurons [ANCOVA, Age^∗^Ganglion, *F*(1, 1) = 0.17, *p* = 0.68; see Table 1]. The mean resting potential of SGN neurons (least-squares adjusted mean) is on average more depolarized than that of VGN neurons (−58.3± 0.90 vs. −61.6 ± 0.65 mV). The SGN data skew toward a younger range with more graded-responding cells than VGN. These two features may partly account for the more depolarized resting potentials of the SGN population.

#### Excitability by Voltage Threshold

**FIGURE 4:**
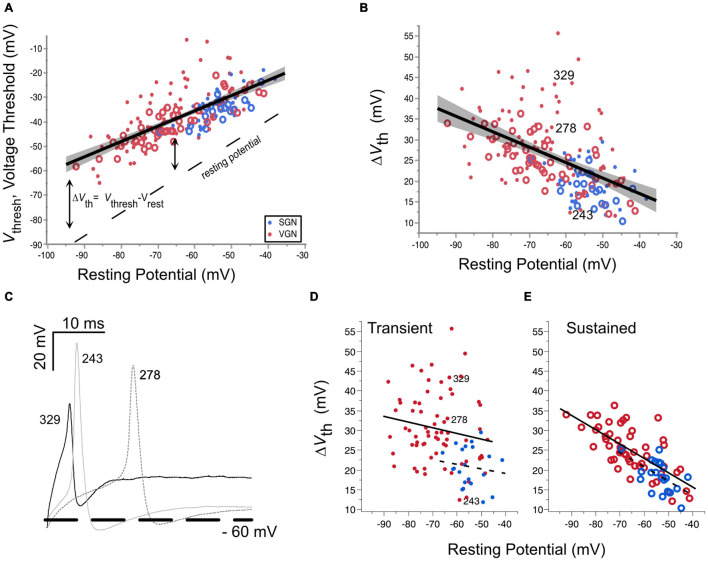
Voltage threshold hyperpolarizes with resting potential in both spiral ganglion and vestibular ganglion. **(A)** Voltage threshold as a function of resting potential in spiral (blue) and vestibular ganglion neurons (red). Sustained-spiking neurons are drawn with open circles, transient-spiking neurons are drawn with small solid circles. Linear regression through all the SGN and VGN data pooled with 95% confidence intervals drawn as a solid line with gray shading. *F*(1, 171) = 171.8, *p* < 0.0001. The dashed line indicates the resting potential. **(B)** Relative voltage threshold (voltage threshold minus the resting potential) plotted as a function of resting potential. *F*(1, 171) = 61.6, *p* < 0.0001. **(C)** Example spike waveforms illustrating the range of voltage thresholds possible for three transient-spiking neurons from VGN. The cells were chosen to have similar resting potentials near –60 mV. The number next to each trace indicates the cell ID. A thick dashed line is drawn at –60 mV. **(D)** Voltage thresholds compared between SGN (dashed regression line) and VGN (solid regression line) for cells with transient-firing patterns (dots). **(E)** Voltage thresholds compared between SGN and VGN for sustained-firing patterns (open circles). Note that sustained-**(A,B)** firing patterns are pooled together. The cell ID indicates the three cells from **(C)**.

Voltage threshold is the minimum voltage needed to produce an action potential. It is mainly dependent on the availability of sodium and calcium channels to open in a positive feedback loop to generate the upstroke of an action potential (Bean, 2007; Platkiewicz and Brette, 2010, 2011). Voltage threshold is not a fixed quantity but can vary from cell to cell and in an individual cell depending on its recent history. In the present data set, the voltage threshold varies anywhere from −65 to −10 mV. This wide range can be partly attributed to the wide range of resting potentials found in the two ganglion groups. The relationship between voltage threshold and resting potential is illustrated in Figure 4. Voltage threshold hyperpolarizes with resting potential (Figure 4, regression for pooled VGN (red symbols) and SGN (blue symbols), *p <* 0.001). Since resting potential depends on age (Figure 3), hyperpolarization of voltage threshold with resting potential is equivalent to hyperpolarization of voltage threshold with postnatal age (*p <* 0.001, regression not shown).

The hyperpolarization of voltage threshold with resting potential is consistent with the idea that the inactivation properties of sodium channels shape voltage threshold. As the resting potential of cells hyperpolarizes, a smaller proportion of the available sodium channels is likely to be inactivated. The greater availability of sodium channels would lower voltage threshold. As described in the modeling of Platkiewicz and Brette (2010), the rate at which voltage threshold changes over a range of membrane potentials depends on the voltage-inactivation range of sodium channels. Voltage threshold becomes most sensitive to membrane potential near the half-inactivation voltage. Conversely, it becomes insensitive to membrane potential at voltages where the sodium channels are wholly relieved from inactivation. In the VGN, the trend for voltage threshold to hyperpolarize persists even at the most negative resting potentials (between −80 and −90 mV). This suggests that the sodium channels in VGN are likely to be partly inactivated even at these more negative resting potentials. Whether this is also true in SGN is not clear since the resting potentials of the SGN did not extend as far negative as in the VGN, presumably due to skew in SGN data toward younger ages when neurons are more depolarized.

An analysis of covariance revealed that the dependence of voltage threshold on resting potential is similar between the two ganglion groups [ANCOVA: Vrest^∗^Ganglion, *F*(1, 1) = 1.85, *p* = 0.175]. This suggests that over a similar range of resting potentials, the average half-inactivation voltage and slope factor for sodium currents is similar between the two ganglion groups.

Although voltage threshold hyperpolarizes with resting potential, this does not mean that the cells are more excitable. This is because the voltage threshold does not change at the same rate as the resting potential (compare the slope of the voltage threshold regression to the dashed line marking the resting potential). Indicating a decrease in excitability, the relative voltage threshold (△*V*_*th*_ = *V*_*thresh*_-*V*_*rest*_) increases as resting potential hyperpolarizes [*F*(1, 171), *p* < 0.001].

△*V*_*th*_ was notably more variable in transient-firing neurons than in sustained-firing neurons (compare the spread in the dots vs. circles in Figure 4B). Figure 4C shows example action potential traces from three transient-spiking neurons (numbers in the figure indicate the cell ID) that were similar in resting potential but had very different voltage thresholds. The difference in the range of △*V*_*th*_ between transient and sustained-firing groups is illustrated by the regressions plotted for each firing type in Figures 4D,E, respectively. When broken down by firing pattern, the least square adjusted mean of △*V*_*th*_ was smaller for SGN than in VGN for transient-spiking neurons (22± 3.5 mV vs. 30±1.0 mV, SGN, and VGN, respectively) but was not different for sustained-spiking neurons (22±1.5 mV vs. 24± 0.6 mV, SGN and VGN, respectively). More data in SGN at older ages is needed to understand if the younger age of SGN accounts for the difference between sustained groups in SGNN and VGN. Another possibility is that the transient-spiking neurons with high thresholds in VGN belong to a distinct subgroup of cells that are considerably larger than any of the other cells in VGN and SGN.

#### Excitability by Current Threshold

**FIGURE 5:**
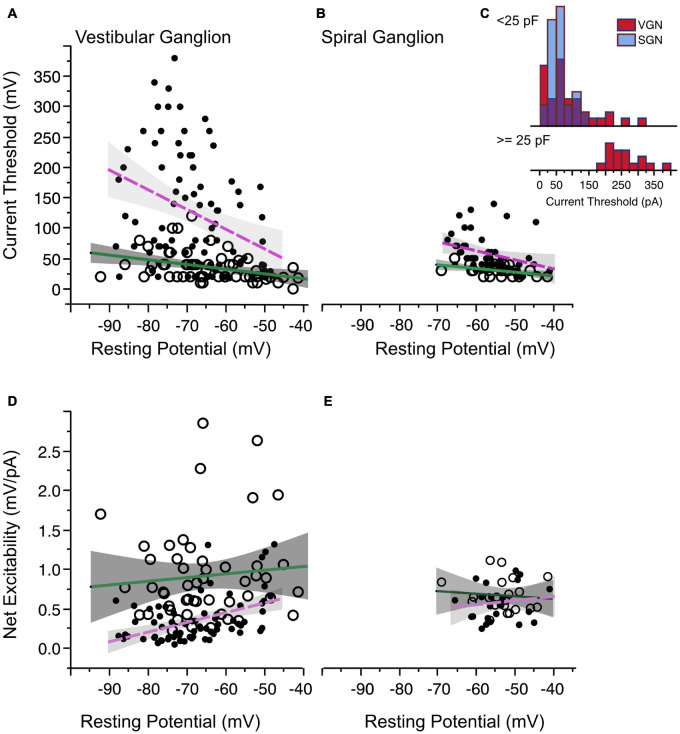
Current threshold and net excitability. **(A)** Current threshold as a function of resting potential in vestibular ganglion neurons. Sustained-spiking neurons are drawn with open circles, transient-spiking neurons are drawn with small solid circles. Lines with gray shading show linear regressions with 95% confidence intervals (dashed magenta line for transient-firing and solid green line for sustained-firing). **(B)** Current threshold vs. resting potential for spiral ganglion neurons. Symbols and regression lines as in **(A)**. **(C)** Histogram of current thresholds in transient spiking neurons. The top histogram is for small to medium-sized cells with capacitance values less than 25 pF. All SGN are assumed to belong to this group. The bottom histogram is for large-sized cells with capacitance values greater than 25 pF. Current thresholds are larger for these cells than for the rest of the SGN and VGN combined. Blue bars indicate SGN and red bars indicate VGN. **(D,E)** The ratio between voltage and current threshold (net excitability) is plotted against resting potential for VGN and SGN.

Figures 5A,B plot the minimum current intensity required to induce spiking from VGN and SGN neurons, respectively. As in Figure 4, the current threshold is plotted against resting potential to evaluate if the variance in this biophysical measure is also related to the maturational change in resting potential. In both VGN and SGN, there is a significant correlation between resting potential and current threshold (*p* = 0.0003 in VGN, *p* = 0.0325 in SGN). Overall, larger stimulus currents are needed to induce spiking in transient-spiking neurons than in sustained-spiking neurons (compare the offset between the magenta and green regression lines in Figures 5A,B). This is consistent with transient-spiking neurons in VGN and SGN having larger low-voltage gated potassium currents., which oppose membrane depolarization (Mo et al., 2002; Kalluri et al., 2010).

One distinct difference between the VGN and SGN is the range of current thresholds observed. Some VGN neurons required as much as 350 pA current injection to generate an action potential. Part of the differences could be due to the SGN dataset being limited to a younger age range where resting potentials are more depolarized and current thresholds are generally smaller in both VGN and SGN. Another more significant factor is that transient firing neurons have a much wider range of cell sizes (refer back to Figure 2L). Figure 5C (inset into Figure 5B) shows that the current thresholds of SGN neurons is similar to that of the small-intermediate sized (< 25 pF) transient neurons from the VGN. The largest current thresholds are from the large VGN neurons (> 25 pF).

The combination of voltage threshold and current threshold determine the net excitability of a neuron. Indicative of their greater net excitability, the ratio between the relative voltage threshold and current threshold was larger in sustained-spiking neurons than in transient-spiking neurons in VGN (Figure 5D in VGN). Although the trend was similar in SGN, it did not reach statistical significance (Figure 5E, effect tests based on ANCOVA summarized in Table 1).

#### The Mutual Dependence of Current Threshold and First-Spike Latency on Input Resistance and Cell Size

**FIGURE 6:**
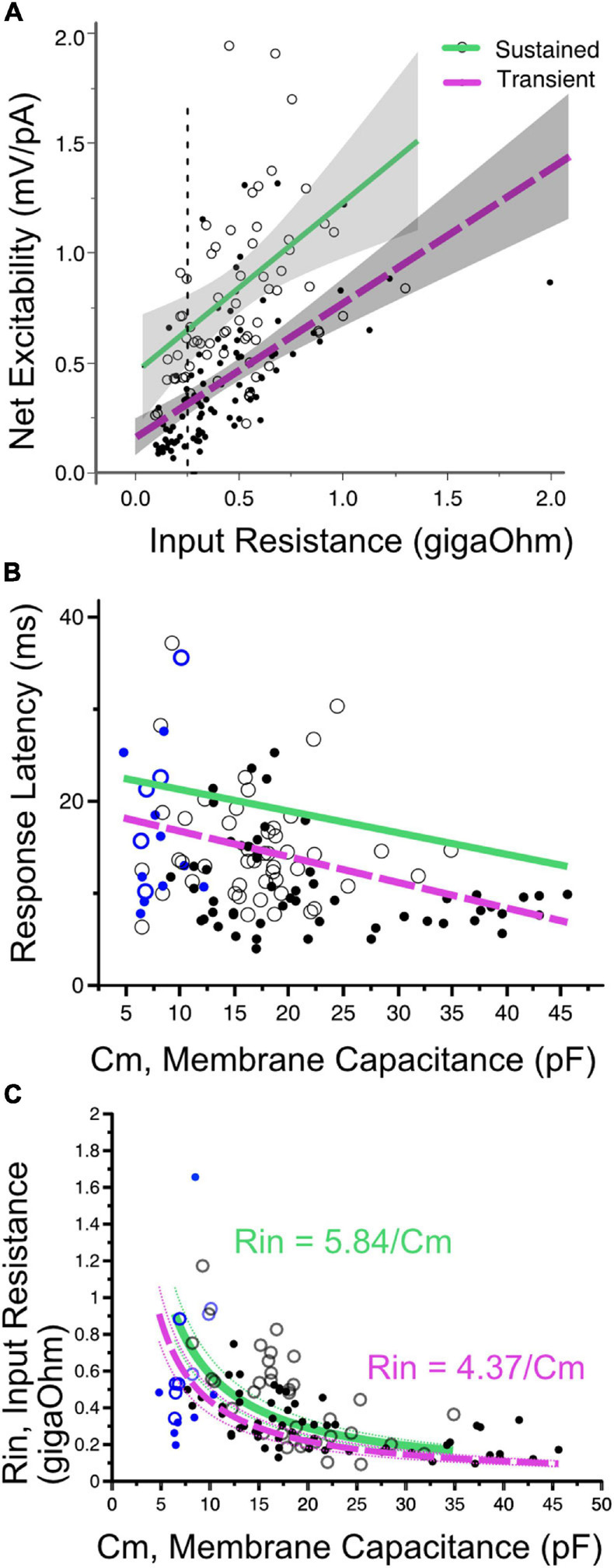
**(A)** Net excitability vs. input resistance for transient- (solid dots) and sustained-spiking neurons (open circles) in VGN and SGN pooled. Regression lines plus 95% confidence intervals are drawn through each firing pattern group. Sustained and transient-spiking neurons with magenta and green lines, respectively. Cells with similar input resistance (e.g., 0.25 gOhms, dotted verticle line) differ in net-excitability, with sustained-spiking neurons being more excitable than transient-spiking neurons. **(B)** Indicative of covariance between input resistance and cell size, first-spike latency decreases with increasing membrane capacitance. Solid magenta and dashed green lines show linear regressions through sustained- and transient-spiking cells. VGN and SGN data are distinguished by black and blue symbols, respectively. Symbols defined as in A. ANCOVA effect tests comparing regressions are shown in Table 1. **(C)** Input resistance decreases with membrane capacitance (*C*_*m*_). The data are fit with two functions in the form *R*_*in*_ = *A*/*C*_*m*_. *A* is equivalent to the specific membrane resistance and is larger for sustained-spiking neurons than for transient spiking neurons. The relationship between *R*_*in*_ and *C*_*m*_ is consistent with the idea that the number of ion channels grows with cell size, but channel density remains approximately constant. The fits are drawn through the sustained and transient-spiking neurons with magenta and green lines, respectively. Dotted lines are 95% confidence intervals. Blue symbols indicate spiral ganglion neurons and black symbols indicate vestibular ganglion neurons.

Not surprisingly, net excitability (which has units of resistance) is positively correlated with input resistance (Figure 6A). However, when cells with the same input impedance are compared, sustained-spiking cells are more excitable than are transient-spiking cells (larger ratio of voltage threshold/current threshold). This means that the difference in excitability between sustained-firing and transient-firing neurons is not just a reflection of differences in resting membrane properties but also dependent on the voltage-gated currents that shape membrane potential between rest and threshold (e.g., *I*_*KL*_).

First-spike latency reflects the temporal integration properties of ganglion neurons. The time to generate a spike is expected to be determined by both the membrane capacitance and input resistance of the cell; membrane time constant (τ = *R*_*in*_*C*_*m*_). When input-impedance is considered on its own (e.g., for cells of uniform size), cells with larger input impedance would take longer to reach voltage threshold, albeit with smaller amplitude current injections. In SGN where cell size is relatively uniform, first-spike latency varies with terminal contact position in inner hair cells; modiolar-contacting SGN have shorter first-spike latencies than pillar-contacting SGN (Markowitz and Kalluri, 2020). Based, in part, on this variation in first spike-latency and uniformity in cell size, Markowitz and Kalluri (2020) concluded that modiolar-contacting SGN have larger net conductance when compared to pillar-contacting SGN.

Suppose cell capacitance was considered on its own. In that case, one might expect first-spike latency to be the longest in the largest cells, but this is not the case (Figure 6B). Some of the fastest first-spike latencies are from the largest cells in the VGN and the longest latencies are small cells from SGN. ANCOVA effect test showing a significant effect of capacitance and firing pattern on latency is reported in Table 1.

The large capacitance of the large somata in VGN is balanced by these cells also having the smallest input resistance. Indeed, cell size and input resistance are covarying. The systematic covariance between input-resistance (*R*_*in*_) and membrane capacitance (*C*_*m*_) is illustrated in Figure 6C. The relationship is fit by a function with the form *R*_*in*_ = *A*/*C*_*m*_. A straightforward interpretation of this relationship is that small and large cells are approximately scaled versions of each other with similar ion channel density per unit area of the membrane (i.e., the specific membrane resistance, *A*, is constant as cell diameter increases). Since input resistance and capacitance scale together, this accounts for why first-spike latency does not increase with cell size. If one views the value of *A* as the specific-membrane resistance, then the lower value of *A* for transient-firing neurons compared to sustained-firing neurons remains consistent with this group of neurons expressing large low-voltage gated potassium conductances (Mo et al., 2002; Iwasaki et al., 2008; Kalluri et al., 2010).

**FIGURE 7:**
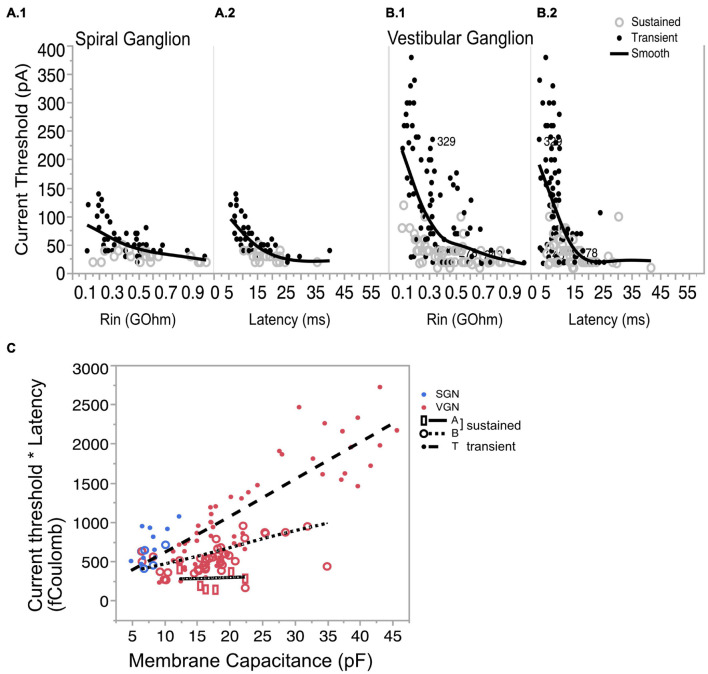
Covariation of current threshold and first-spike latency. **(A.1)** Current threshold decreases with input resistance for sustained- and transient-spiking neurons in SGN. **(A.2)** Current threshold as a function of response latency showing that cells with large thresholds are also the cells with short latencies. **(B.1,B.2)** Same as **(A.1,A.2)** but for VGN. Solid lines in **(A.1,A.2,B1,B.2)** are smoothed spline curves drawn through the pooled transient- and sustained-spiking groups. **(C)** Charge (*Q*) that is required to reach action-potential peak plotted as a function of membrane capacitance. *Q* is estimated by multiplying (*) the current threshold by the first-spike latency. Symbols with regression lines indicate transient-spiking (dots/dash line), sustained-B spiking (open circles/dotted line) and sustained-A spiking (open rectangles/solid line). Spiral ganglion neurons are indicated in blue symbols and vestibular ganglion neurons are indicated by red symbols. * indicated multiplication of the two quantities.

By Ohm’s Law, the amount of current needed to effect a fixed change in voltage is expected to increase with input resistance (Figure 7A.1 for SGN and Figure 7B.1 for VGN). Related by their mutual dependence on input resistance, current threshold is covariant with first-spike latency (*L*) in both SGN and VGN (Figure 7A.2 for SGN and Figure 7B.2 for VGN). Another way to express the excitability of these ganglion neurons is to look at the minimum charge delivered to excite an action potential (*Q*). Multiplying the current threshold by the first spike latency (*Q* = *I*_*threshold*_^∗^*L*) roughly estimates the charge. Since the stimulus is a current step, this value is equivalent to the area under the stimulus from onset of the current step to the first-spike. *Q* depends on both cell size and firing pattern (Figure 7C). For any fixed cell size, the sustained-A group requires the smallest charge transfer to trigger spiking. This was followed by sustained-B and then transient-spiking neurons. If we consider all SGN as small cells (including cells without capacitance measurements), transient-firing SGN require significantly more charge to trigger spiking than do sustained-B firing SGN (758 ± 3, *n* = 46 vs. 546 = /−44 *n* = 24; *t*-test, df = 66, *p* < 0.001).

The systematic dependence of *Q* on cell size and firing pattern group reveals an inherent structure to the biophysical heterogeneity seen across the two ganglia. As the regression lines in Figure 7C show, *Q* is sensitive to cell size, but this sensitivity differs between firing pattern groups; note that the slopes of the regression lines are steeper for the transient and sustained-B cells than for the sustained-A cells. Indeed, *Q* is relatively invariant to cell size in the sustained-A firing group. The relative insensitivity to cell size is consistent with the idea that sustained-A neurons have the largest specific membrane resistance and thus have the slowest rate of change in input resistance with cell size. In contrast, *Q* is sensitive to cell size in sustained-B neurons and most sensitive to cell size in transient-spiking neurons. The dependence of *Q* on cell size reflects the neurons’ inherent sensitivity to depolarization rate. The steady-state activation of low-voltage gated channels means that transient-firing neurons have low input impedance, which requires larger currents to depolarize the neuron to voltage threshold. The activation kinetics of the potassium currents sets a time limit within which the membrane potential must reach spike threshold.

## Discussion

### Spiral Ganglion Neurons and Vestibular Ganglion Are Part of the Same Biophysical Continuum

Heterogeneity in the ion channel properties of spiral and vestibular ganglion has long been evident in the diversity of firing patterns observed in response to current injections. Many *in vitro* studies in both sensory systems have described this diversity independently; revealing qualitative similarities in firing patterns and in the types of ion channels responsible for shaping the firing patterns (Mo and Davis, 1997a; Mo et al., 2002; Risner and Holt, 2006; Iwasaki et al., 2008; Hight and Kalluri, 2016). Indicative of the overall heterogeneity in their ion channel composition, our combined analysis of vestibular and spiral ganglion neurons revealed a wide range of firing patterns, resting potentials, voltage thresholds, current thresholds, and first-spike latencies.

Transient-spiking and sustained-spiking patterns were found in both ganglion groups. The biophysical distinctions between these two firing pattern groups neurons are qualitatively similar across the two ganglia. For example, transient-firing neurons were less excitable than sustained-spiking neurons in both SGN and VGN. These differences are consistent with previous patch-clamping studies in each system, showing that the presence of low-voltage gated potassium currents (e.g., conducted through Kv1 and KCNQ channels) is responsible for spike-train accommodation (Mo et al., 2002; Iwasaki et al., 2008; Kalluri et al., 2010). The role of low-voltage gated potassium currents in shaping firing patterns and excitability is not unique to the inner ear ganglion neurons. Similar firing pattern groups have also been described in auditory brainstem neurons (Oertel, 1983; Rothman and Manis, 2003). Bushy and Octopus cells are both known to have low impedance and produce transient-firing patterns because they contain large low-voltage gated potassium currents. In contrast, stellate cells either lack or have smaller potassium currents to produce more sustained responses.

In both SGN and VGN, the prevalence of ganglion neurons with sustained-A firing patterns decreased with postnatal age. Developmental changes in firing patterns were accompanied by an overall hyperpolarization in resting potential, a decrease in input resistance, and an increase in current threshold. The covariances between biophysical properties such as resting potential and voltage threshold and their dependence on postnatal age were similar between the two ganglia. For example, resting potential changed with postnatal age and voltage threshold changed with resting potential. Notably, the rate of hyperpolarization in resting potential and voltage threshold was statistically similar for spiral and vestibular ganglion neurons. These developmental trends are consistent with previous electrophysiological studies showing that the density of low-voltage gated potassium currents (which regulate spike patterns and resting potentials) increase during maturation (reviewed in Davis and Crozier, 2015 for SGN; Ventura and Kalluri, 2019 for VGN). Consistent with electrophysiology, recent RNA sequencing results in SGN show enrichment of potassium channels during postnatal maturation (Shrestha et al., 2018; Sun et al., 2018; Petitpré et al., 2018). Similarly, immunohistochemistry and pharmacology in VGN provide evidence for a developmental upregulation in low-voltage gated potassium currents and their underlying channels (Rennie and Streeter, 2006; Pérez et al., 2009; Kalluri et al., 2010; Meredith and Rennie, 2015). For example, KCNQ channels (which carry part of low-voltage gated potassium currents) are initially confined to a subset of neurons innervating the central/striolar zones of the vestibular sensory epithelia. The expression pattern spreads to include peripheral/extrastiolar zones during postnatal development (Hurley et al., 2006). By P21, nearly all VGNs in mice immunolabel for KCNQ channels (Rocha-Sanchez et al., 2007). Developmental upregulation of these channels can account for the hyperpolarization of resting potentials and the tendency for firing patterns to become more phasic transient- and sustained-B firing patterns.

Although there are significant qualitative similarities in the biophysical properties of VGN and SGN, there are quantitative differences. In most of the biophysical measures, VGN have a broader range of values than in the SGN. This included more hyperpolarized resting potentials, a wider range of voltage thresholds, a larger range of current thresholds, and more diversity in cell size as probed by membrane capacitance in VGN than in SGN. This may partly be because the VGN data extend to older ages than the SGN. Since there are biophysical changes due to developmental in both neuronal groups, this likely contributes to some of the absolute differences in biophysical properties. Differences in cell size likely contribute to biophysical differences between SGN and VGN. Based on the membrane capacitance, spiral ganglion neurons are small and relatively homogenous in cell size, whereas vestibular ganglion neurons are more heterogenous with a bi-modally distribution in cell sizes. The electrophysiological estimates of cell sizer are consistent with previous morphological analyses; SGN somata have relatively homogenous cross-sectional areas (e.g., Nayagam et al., 2011) when compared to VGN, which are more variable in somatic size (Kevetter and Leonard, 2002).

The electrophysiological properties of large-bodied VGN stood out as being distinct from the rest of the VGN and SGN. These cells had the largest current thresholds, low input impedance, very fast first spike latencies, and fired predominantly with transient-spiking. Even though the biophysical features of these large VGN stand out as being distinct from SGN, the analysis presented here suggests that the large VGN are size-scaled versions of smaller transient-firing neurons in SGN and VGN. For example, the larger current thresholds and faster first spike latencies in the large VGN neurons make sense if the input resistance changes as expected when cell diameter increases while holding channel densities constant. Related by cell size, the biophysical properties of SGN and VGN can be thought of as belonging to the same size-scaled biophysical continuum.

### Limitations

One advantage of the present comparison between SGN and VGN is that the recordings were made under similar conditions (internal solution composition, recording protocols, and enzymatic treatment), and uniform criteria were applied to quantify the biophysical properties of each neuron. However, there are methodological differences that are important to consider. First, SGN recordings are skewed to a younger age range because the dissection for the semi-intact preparation becomes more difficult as the otic capsule ossifies with maturation. In contrast, because the VGN were disassociated and cultured, the dataset extends to older ages. Second, the difference between acute and cultured preparations means that the somatic membranes may be in different states. Previous work has shown that time in culture and culture conditions have can impact firing patterns (Adamson et al., 2002; Zhou et al., 2005; Sun and Salvi, 2009). This may be why there is a greater prevalence of graded-firing neurons in the acute SGN than in cultured VGN. Indeed, graded responses are reported in other studies where SGN were prepared without a significant period in culture (Santos-sacchi, 1993; Jagger and Housley, 2003). This may be because ion channels redistribute to portions of the membrane otherwise covered by myelin in the endogenous condition (as described in CNS by Shrager, 1993).

### Which Ion Channel Combinations Are Driving Biophysical Diversity?

In both the spiral and vestibular ganglion, similar groups of ion channels are thought to be important for shaping firing patterns (Mo et al., 2002; Liu and Davis, 2007; Iwasaki et al., 2008; Kalluri et al., 2010; Yoshimoto et al., 2015). Our previous work and that of many others have focused on currents conducted by low-voltage gated potassium channels (*I*_*KL*_), like Kv1 and KCNQ, and currents conducted by hyperpolarization-activated cyclic-nucleotide gated channels, HCN (*I*_*H*_). These currents are powerful modulators of neuronal activity because they have voltage-gated properties that make the currents available between rest and threshold. They can influence cell excitability by regulating resting potential, input resistance, membrane integration times, and firing patterns.

Based on pharmacology and modeling, spike-train accommodation is regulated by the density of *I*_*KL*_ (e.g., Mo et al., 2002; Iwasaki et al., 2008; Kalluri et al., 2010; Hight and Kalluri, 2016). *I*_*KL*_ prevents repetitive spiking because activation between rest and threshold prevents the cell from recovering to threshold after the first spike. Because *I*_*KL*_ is typically active at resting potential, cells having these currents tend to have hyperpolarized resting potential and lower input resistance. Ultimately, in neurons that express large *I*_*KL*_, the race between reaching threshold and the activation of *I*_*KL*_ makes these neurons sensitive to the rate of voltage depolarizations. As a result, cells with large *I*_*KL*_ respond to the sharp onsets of step stimuli in response to current steps but not to the sustained portion of the stimulus.

Voltage threshold can be regulated by the type, density, and inactivation properties of sodium channels and by the presence of potassium/leak channels that counter the depolarization force of sodium channels (Platkiewicz and Brette, 2010, 2011). As a result, voltage threshold can vary from cell to cell and depend on a cell’s history. Here the more negative the resting potential of a cell, the more hyperpolarized the voltage threshold. This can broadly be understood as resulting from fewer sodium channels being inactivated at rest for cells with more hyperpolarized resting potentials. According to the modeling by Platkiewicz and Brette (2011), voltage threshold is likely to be most sensitive to membrane voltage near the half-inactivation voltage for sodium currents. The finding voltage threshold is similarly dependent on resting potential in VGN and SGN is consistent with the idea that the sodium currents are primarily carried by TTX sensitive transient Nav1.6 channels (Hossain, 2005; Lysakowski et al., 2011).

Despite the broad similarity in the threshold’s dependence on resting potential, there is also significant cell to cell variability in threshold, suggesting that variations in sodium channel density and sub-unit composition could further regulate threshold. For example, VGN neurons express various sodium channels, including Nav 1.5 and Nav 1.8 (Liu et al., 2016) and sodium-dependent potassium channels (e.g., Reijntjes et al. (2019) in SGN) which can impact voltage threshold. The variable expression for Nav1.8 in calretinin positive vs. calretinin negative vestibular ganglion neurons provides evidence for differences in sodium channels composition across different cell groups (Liu et al., 2016). Recent analysis in vestibular calyx recordings also suggests that there are zonal and maturational differences in sodium current properties, with peripheral zone neurons having larger resurgent sodium currents which may make those neurons more excitable (Meredith and Rennie, 2020).

Voltage thresholds are more variable in transient-spiking vestibular neurons than in sustained-spiking neurons. A similar difference between firing pattern groups was previously noted in spiral ganglion, where neurons that fired only a single action potential had the most varied and depolarized voltage thresholds (Crozier and Davis, 2014). In addition to sodium channels, Kv1 and HCN channels, which are known to be important for transient firing, are known to regulate voltage threshold in SGN (Liu et al., 2014). Given the similar roles played by Kv1 and HCN channels in transient-firing neurons, it seems highly likely that they are similarly impacting voltage threshold in VGN, but this remains to be directly tested.

### Relating Biophysical Diversity to Functional Diversity

On the surface, it seems peculiar that auditory and vestibular neurons, each with such diverse morphologies and functions, share so many common traits. Many of the individual components of the afferent synapses are similar in the two systems. For example, the synapses between hair cells and afferent neurons are composed of electron-dense ribbon-bearing pre-synaptic active zones that drive post-synaptic glutamate receptors. Across many studies, gradients in the same two classes of ionic currents (low-voltage gated potassium currents, *I*_*KL*_ and hyperpolarization-activated cationic currents, *I*_*h*_) of the post-synaptic neurons are proposed as being crucial to diverse neuronal functions (Mo and Davis, 1997a,b; Mo et al., 2002; Risner and Holt, 2006; Kalluri et al., 2010; Yi et al., 2010; Horwitz et al., 2011; Almanza et al., 2012). The analysis here shows that the biophysical properties of the post-synaptic neurons are not just qualitatively similar but can be quantitatively related.

Neurons with large *I*_*KL*_ having low-input resistance, large current thresholds, fast membrane time constants, and sensitivity to rate of depolarization have also been described in the timing pathways of the auditory brainstem (Bal and Oertel, 2001; Ferragamo and Oertel, 2002; Rothman and Manis, 2003). There the presence of *I*_*KL*_ is critical for coincidence detection (reviewed in Golding and Oertel, 2012). Similarly, in vestibular afferents, the short temporal integration time in the presence of *I*_*KL*_ drives *in vitro* neurons to produce irregular spiking by allowing them to respond preferentially to rapid and randomly timed changes in simulated synaptic current (Kalluri et al., 2010). In contrast, *in vitro* ganglion neurons lacking *I*_*KL*_ (possibly combined with HCN mediated currents) have long integration times to smooth the rapid fluctuations in the incoming stimulus to generate highly regular spiking. These ideas about the possible impact of biophysical diversity on afferent responses are based on *in vitro* experiments where the somata lack normal synaptic input, dendritic filtering and efferent modulation. To make definitive associations between *in vitro* and *in vivo* patterns will require future work to test if somata with distinct firing patterns belong to afferent neurons with distinct morphology or patterns of spatial connectivity within the epithelium.

Consistent with the idea that *in vitro* neuronal classes can be related to *in vivo* classes, the large, transient-firing somata in the vestibular ganglion likely belong to the irregular-firing afferents innervating the central zones of vestibular epithelia. This is based on the observation (based on molecular expression patterns) that the somata of irregularly firing afferents from central epithelial zones tend to be larger than that of regular-firing vestibular afferents (Leonard and Kevetter, 2002). Like the large-bodied somatata, calyx terminals in striolar zone mouse vestibular epithelia produce transient-spiking and express large Kv1 and KCNQ channels when compared to extrastriolar calyces (Songer and Eatock, 2013). The morphology of pure-calyx bearing neurons suggests that these neurons are designed to respond to large, temporally compact inputs; this is the type of synaptic input one might expect from the synchronous release of neurotransmitters from many synaptic ribbons driven by small groups (1–3) of closely spaced hair cells. The arrangement of the hair cells and their ribbon synapses suggests that they might produce highly synchronized fast onset synaptic events such as those predicted by Holmes et al. (2017) to drive irregular spiking.

In contrast to the diverse range of dendritic morphologies found in vestibular afferents, the morphology of auditory afferents is homogenous. Liu and Davis (2007) proposed the idea intrinsic diversity in voltage thresholds contributes to functional diversity I the auditory nerve (i.e., diversity in sound-intensity threshold and spontaneous rates across auditory neurons). Recent work in spiral ganglion shows that the biophysical diversity and transcriptomic diversity of ganglion neurons aligns (Petitpré et al., 2018; Shrestha et al., 2018; Sun et al., 2018; Markowitz and Kalluri, 2020) with a previously described spatial map for spontaneous rates and sound- intensity thresholds (Liberman, 1982). The somata of auditory nerve fibers contacting the pillar face of inner hair cells (putative high-spontaneous rate fibers) have lower current thresholds, higher-input impedance, and longer first-spike latencies compared to the somata of fibers contacting the modiolar face of the inner hair cell (putative low-spontaneous rate fibers) (Markowitz and Kalluri, 2020). Differences in temporal integration could contribute to the response of SR-groups by making neurons selective to the different kinetics of synaptic current. For example, synaptic events at inner hair cells are characterized by a combination of multi-phasic slow-onset events and monophasic fast-onset events (Grant et al., 2010). With shorter synaptic integration windows (reflected in first-spike latency) and lower input impedance, low-SR fibers would need large, fast onset currents to reach threshold. In contrast, the longer integration windows and lower current thresholds of pillar-contacting fibers could support faster spike rates by allowing the neuron to respond to a greater variety of synaptic inputs; e.g., both mono-phasic and multi-phasic events.

Diversity in intrinsic biophysics is only one of several factors that combine to produce an afferent neuron’s response. This means that neuronal membranes with similar intrinsic biophysical properties could produce very different responses depending on the input. As schematized in Figure 1, the bipolar neurons of auditory and vestibular nerves are diverse in many ways. They vary in the type and number of hair cells providing input to the neurons. The temporal features of synaptic input are likely to be complex, depending on the makeup of the hair cells providing input to hair cells. For example, vestibular hair cells transmit information through both chemical and non-chemical mechanisms (Songer and Eatock, 2013; Sadeghi et al., 2014; Contini et al., 2019). Moreover, the complex dendritic arbors of vestibular neurons are likely to filter synaptic input differently than the unitary short dendrite of auditory afferents. Models of vestibular neuron activity predict that regular- and irregular-timed spiking is determined both by the intrinsic biophysical properties of the neuron and by the nature of the synaptic input (Smith and Goldberg, 1986; Hight and Kalluri, 2016). For example, modeled sustained-A neurons are equally capable of producing regular and irregular firing depending on how much of the temporal fluctuations in the synaptic signals are allowed through to the spike initiation zone (Hight and Kalluri, 2016). Future work will need to consider how dendritic morphology, synaptic specializations, and ion channel properties combine to produce functionally distinct neuronal subgroups in the auditory and vestibular nerve.

## Data Availability Statement

The original contributions presented in the study are included in the article/supplementary material, further inquiries can be directed to the corresponding author/s.

## Ethics Statement

The animal study was reviewed and approved by the Institutional Animal Care and Use Committee at the University of Southern California.

## Author Contributions

RK: conceptualization, resources, data curation, software, formal analysis, supervision, funding acquisition, validation, investigation, visualization, methodology, project administration, writing—original draft, and editing.

## Conflict of Interest

The author declares that the research was conducted in the absence of any commercial or financial relationships that could be construed as a potential conflict of interest.

## Publisher’s Note

All claims expressed in this article are solely those of the authors and do not necessarily represent those of their affiliated organizations, or those of the publisher, the editors and the reviewers. Any product that may be evaluated in this article, or claim that may be made by its manufacturer, is not guaranteed or endorsed by the publisher.
